# Multiplatform metabolomic analysis of the R6/2 mouse model of Huntington’s disease

**DOI:** 10.1002/2211-5463.13285

**Published:** 2021-09-14

**Authors:** Masayo Hashimoto, Kenichi Watanabe, Kan Miyoshi, Yukako Koyanagi, Jun Tadano, Izuru Miyawaki

**Affiliations:** ^1^ Preclinical Research Unit Sumitomo Dainippon Pharma Co., Ltd Osaka Japan; ^2^ Pharmacology Research Unit Sumitomo Dainippon Pharma Co., Ltd Osaka Japan

**Keywords:** Huntington's disease, metabolomics, R6/2 mouse

## Abstract

Huntington’s disease (HD) is a progressive, neurodegenerative disease characterized by motor, cognitive, and psychiatric symptoms. To investigate the metabolic alterations that occur in HD, here we examined plasma and whole‐brain metabolomic profiles of the R6/2 mouse model of HD. Plasma and brain metabolomic analyses were conducted using capillary electrophoresis–mass spectrometry (CE‐MS). In addition, liquid chromatography–mass spectrometry (LC‐MS) was also applied to plasma metabolomic analyses, to cover the broad range of metabolites with various physical and chemical properties. Various metabolic alterations were identified in R6/2 mice. We report for the first time the perturbation of histidine metabolism in the brain of R6/2 mice, which was signaled by decreases in neuroprotective dipeptides and histamine metabolites, indicative of neurodegeneration and an altered histaminergic system. Other differential metabolites were related to arginine metabolism and cysteine and methionine metabolism, suggesting upregulation of the urea cycle, perturbation of energy homeostasis, and an increase in oxidative stress. In addition, remarkable changes in specific lipid classes are indicative of dysregulation of lipid metabolism. These findings provide a deeper insight into the metabolic alterations that occur in HD and provide a foundation for the future development of HD therapeutics.

## Graphical abstract

Multiplatform metabolomic analysis revealed several perturbed metabolic pathways in the R6/2 mouse model of Huntington’s disease. Altered histidine metabolism in the brain indicates neurodegenerative status and an altered histaminergic system. Changes in the metabolism of arginine, cysteine, and methionine, and various lipid species were also highlighted. These findings add deeper insights into the metabolic alterations in Huntington’s disease.

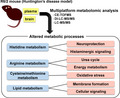

**Abbreviations**
CE‐TOFMS: capillary electrophoresis time‐of‐flight mass spectrometry
DI‐LC‐MS/MS: direct injection liquid chromatography–tandem mass spectrometry
FIA‐MS/MS: flow injection analysis tandem mass spectrometry
HD: Huntington’s disease
PCA: principal component analysis
WT: wild type

Huntington’s disease (HD) is a progressive, neurodegenerative disease characterized by motor, cognitive, and psychiatric symptoms. HD is caused by an expanded CAG repeat in the huntingtin gene, which encodes a polyglutamine stretch in the huntingtin protein [1]. Although various molecules interacting with mutant huntingtin have been described, molecular and cellular mechanisms underlying the pathogenesis of HD have not been fully elucidated. There is no disease‐modifying treatment which can arrest or reverse the progression of HD, and novel pathophysiological insights and therapeutic approaches are needed. A number of genetically modified animal models of HD have been generated. Among these models, the R6/2 mouse is the most widely used animal model for HD and displays many signs and symptoms similar to those of clinical HD, such as neuronal intranuclear inclusions, cognitive deficits, and locomotor disturbances [2, 3, 4]. The R6/2 mouse demonstrates motor deficits beginning at 6 weeks of age. The motor deficits are progressive and followed by early death at 12–15 weeks of age [2, 5, 6].

Metabolomics is a technology to comprehensively characterize the metabolites in biological systems. In recent years, the metabolomic approach has been increasingly used to understand disease mechanisms, discover new biomarkers for disease diagnoses, and identify novel drug targets [7]. Since metabolites in biological system have various physical and chemical properties, metabolomic investigations require powerful analytical strategies. Nuclear magnetic resonance spectroscopy (NMR), gas chromatography–mass spectrometry (GC‐MS), liquid chromatography–mass spectrometry (LC‐MS), and capillary electrophoresis–mass spectrometry (CE‐MS) are powerful and widely used analytical techniques in metabolomics studies [8, 9]. Each analytical technique has its own advantage points; for instance, CE‐MS is a robust technique suitable for highly polar and charged metabolite analyses, while LC‐MS is widely used in hydrophobic metabolites and lipid analyses. Therefore, the combination of these techniques enables to cover the broad range of metabolites in complex biological samples.

Previous metabolomic studies of HD patients as well as animal models have demonstrated several metabolic alterations, such as changes in protein metabolism, carbohydrate metabolism, and cholesterol metabolism [10, 11, 12]. However, these results are varied and inconsistent, and there is still a lack of promising metabolomic biomarkers or therapeutic targets that can be used for exploring new HD therapeutics. In addition, many of previous works of metabolomics on HD applied single‐platform approach, which is difficult to cover the wide range of metabolites with different physiochemical properties.

In this study, we investigated the metabolite profiles of R6/2 mice to improve the understanding of the metabolic alteration in HD. Plasma and whole‐brain metabolome of R6/2 mice at 8 weeks and 10 weeks of age, both are disease progression stages and frequently used in preclinical drug testing [6, 13, 14], were compared with those of wild‐type (WT) littermates. To cover the broad range of metabolites, we applied multiple analytical techniques using CE‐MS and LC‐MS to plasma metabolomic analysis. We also applied metabolomic techniques using CE‐MS to the brain, which is the most pathologically affected tissue in HD.

## Materials and methods

### Animals and sample collection

Female R6/2 mice (B6CBA‐Tg[HDexon1]62Gpb/3J) [2] and WT mice at 3 weeks of age were obtained from Charles River Laboratories Japan (Yokohama, Japan). The R6/2 mouse is transgenic for the 5' end of the human HD gene carrying around 120 CAG repeat expansions. Mice were housed in plastic cages under a 12‐h light–dark cycle at 23 ± 3 °C with free access to standard diet and water. Plasma and whole brain were collected from R6/2 mice and WT mice at 8 and 10 weeks of age (*n* = 5 each). On the sampling day, mice were fasted for approximately three hours before sampling. After the anesthetization with isoflurane, blood was collected from the heart in tubes containing EDTA as anticoagulant, and plasma was obtained by centrifugation. After the blood collection, brain samples were collected. Plasma and brain samples were stored at −80 °C until analysis. All procedures of animal experiments described in this study were approved by the Experimental Animal Welfare Committee of Sumitomo Dainippon Pharma Co., Ltd.

### Chemicals and reagents

Internal standards for LC‐MS/MS analysis, 2‐morpholinoethanesulfonic acid and 12S‐hydroxy‐5Z,8Z,10E,14Z‐eicosatetraenoic‐5,6,8,9,11,12,14,15‐d8 acid (12S‐HETE‐d8), were obtained from Dojindo (Kumamoto, Japan) and Cayman Chemical (Ann Arbor, MI, USA), respectively. The MxP® Quant 500 kit was obtained from Biocrates Life Sciences AG (Innsbruck, Austria). All solvents used for MS were of high‐performance liquid chromatography grade.

### Metabolomic analysis

#### Capillary electrophoresis time‐of‐flight mass spectrometry (CE‐TOFMS) analysis

Plasma and brain sample analyses using CE‐TOFMS were conducted at Human Metabolome Technologies (HMT), Tsuruoka, Japan. The analysis was performed using the Agilent CE‐TOFMS system (Agilent Technologies, Waldbronn, Germany) with methods developed by Soga *et al*. [15, 16, 17]. Briefly, 50 µL of plasma was mixed with methanol containing internal standards (Solution ID: H3304‐1002, HMT) and ultrapure water. An aliquot of the mixtures was ultrafiltered using a 5‐kDa cutoff centrifugal filter (UltrafreeMC‐PLHCC, HMT). The filtrate was evaporated to dryness, and the residue was dissolved in ultrapure water for CE‐TOFMS analysis. Whole‐brain samples were homogenized with 50% acetonitrile containing internal standards and the brain homogenates were centrifuged; then, the supernatants were ultrafiltered using 5‐kDa cutoff centrifugal filters. The filtrate was evaporated to dryness and the residue was dissolved in ultrapure water for CE‐TOFMS analysis. Data obtained by CE‐TOFMS were processed by MasterHands (Keio University, Tsuruoka, Yamagata, Japan) to extract peak information including m/z, peak area, and migration time (MT). Signal peaks corresponding to isotopomers, adduct ions, and other product ions of known metabolites were excluded. The remaining peaks were annotated according to the HMT metabolite database based on their m/z values with the MTs. The tolerance range for the peak annotation was configured at ±0.5 min for MT and ±10 ppm for m/z. The areas of the annotated peaks were then normalized based on internal standard levels.

#### Direct injection liquid chromatography–tandem mass spectrometry (DI‐LC‐MS/MS) analysis (MxP® Quant 500 kit)

Plasma samples were also analyzed using the MxP® Quant 500 kit (Biocrates Life Sciences AG, Innsbruck, Austria). The assay covers 630 metabolites and lipids: 13 small molecule classes (including amino acids and related metabolites, bile acids, amines, carboxylic acids, fatty acids) analyzed with LC‐MS/MS, and 12 lipid classes (including acylcarnitines, phosphatidylcholines, sphingomyelins, ceramides, cholesteryl esters, diacylglycerols, triacylglycerols) and hexoses analyzed with flow injection analysis tandem mass spectrometry (FIA‐MS/MS). Plasma samples were processed and analyzed according to the manufacturer’s instructions. Briefly, 10 µL of plasma was added to the upper chambers of a 96‐well plate and dried in a nitrogen evaporator. Subsequently, a solution of 5% phenylisothiocyanate was added for derivatization of the amino acids and amines. After incubation, the metabolites were extracted using 5 mm ammonium acetate solution in methanol. The extracts were diluted for FIA‐MS/MS analysis and LC‐MS/MS analysis. The analyses were carried out using a Nexera™ X2 high‐performance liquid chromatography (HPLC) system (Shimadzu Co., Kyoto, Japan) and a 6500QTRAP mass spectrometer (AB Sciex, Framingham, MA, USA).

In LC‐MS/MS analyses, a reversed‐phase column (a part of MxP® Quant 500 kit) was used for chromatographic separation. For mobile phases A and B, 2% formic acid in water and 2% formic acid in acetonitrile were used, respectively. The flow rate was 0.8 mL·min^−1^ (0–4.7 min) or 1.0 mL·min^−1^ (4.7–5.8 min). The column oven temperature was set at 50 °C. For LC‐MS/MS‐positive mode analysis, the gradient of mobile phase B concentration was programmed as 0% (0 min) – 0% (0.25 min) – 12% (1.5 min) – 17.5% (2.7 min) − 50% (4 min) − 100% (4.5 min) − 100% (5 min) − 0% (5.1 min) − 0% (5.8 min). For LC‐MS/MS negative mode analysis, the gradient of mobile phase B concentration was programmed as 0% (0 min) – 0% (0.25 min) – 25% (0.5 min) – 50% (2 min) – 75% (3 min) − 100% (3.5 min) − 100% (5 min) − 0% (5.1 min) − 0% (5.8 min). The mass spectrometer was operated using an electrospray ionization source in positive or negative mode. The parameters for the mass spectrometer were set as follows: ion spray voltage, 5500 V (positive mode) or −4500 V (negative mode); ion source heater temperature, 500 °C (positive mode) or 650 °C (negative mode); nebulizer gas, 60 psi (positive mode) or 40 psi (negative mode); turbo gas, 70 psi (positive mode) or 40 psi (negative mode); curtain gas, 45 psi (positive mode) or 35 psi (negative mode).

In FIA‐MS/MS analyses, flow rate was set as 30 µL·min^−1^ (0 min) − 30 µL·min^−1^ (1.6 min) − 200 µL·min^−1^ (2.4 min) − 200 µL·min^−1^ (2.8 min) − 30 µL·min^−1^ (3 min). The mass spectrometer was operated using an electrospray ionization source in positive mode. The parameters for the mass spectrometer of two FIA methods were set as follows: ion spray voltage, 5500 V (FIA methods 1 and 2); ion source heater temperature, 200 °C (FIA methods 1 and 2); nebulizer gas, 40 psi (FIA method 1) or 30 psi (FIA method 2); turbo gas, 50 psi (FIA method 1) or 80 psi (FIA method 2); curtain gas, 30 psi (FIA method 1) or 20 psi (FIA method 2).

Data were generated using Analyst (AB Sciex, Framingham, MA, USA) software and transferred to MetIDQ™ (Biocrates Life Sciences AG) software for further data processing and the technical validation.

### LC‐MS/MS analysis

A series of primary metabolites and lipid mediators in plasma were analyzed using an LC‐MS/MS system consisting of a Nexera™ X2 HPLC system and a triple quadrupole mass spectrometer LCMS‐8060 (Shimadzu Co., Kyoto, Japan). The analytical methods were set up using LC/MS/MS Method Packages for primary metabolites and lipid mediators (Shimadzu Co.). Each LC/MS/MS Method Package provides optimized analytical conditions including chromatogram acquisition, detection of mass spectral peaks using an incorporated mass spectral library, and their chromatographic data processing for 98 primary metabolites and 160 lipid mediators.

For primary metabolites analysis, the plasma sample (10 µL) was mixed with 100 µL of methanol containing internal standard (2‐morpholinoethanesulfonic acid, 10 µm). After centrifugation, the supernatant (50 µL) was mixed with water (200 µL) and chloroform (200 µL) and centrifuged. The aqueous phase (20 µL) was evaporated to dryness, and the residue was dissolved in 0.1% acetic acid (40 µL) prior to LC‐MS injection (10 µL). A reversed‐phase column (discovery HS F5‐3, 2.1 × 150 mm, 3 μm, Sigma‐Aldrich, St Louis) was used for chromatographic separation. For mobile phases A and B, 0.1% formic acid in water and 0.1% formic acid in acetonitrile were used, respectively. The flow rate was 0.25 mL·min^−1^. The column oven temperature was set at 40°C. The gradient of mobile phase B concentration was programmed as 0% (0 min) − 0% (2 min) − 25% (5 min) −35% (11 min) − 95% (15 min) − 95% (20 min) − 0% (20.1 min) − 0% (25 min). Mass spectrometer parameters for positive/negative electrospray ionization mode were as follows: drying gas flow rate, 10 L·min^−1^; nebulizer gas flow rate, 3 L·min^−1^; desolvation line temperature, 250 °C; interface temperature, 300°C; heat block temperature, 400 °C. The compounds were identified using the LC‐MS/MS method package for primary metabolites and LabSolutions Insight software (Shimadzu Co.). The peak height of each compound was calculated and normalized to the peak height of the internal standard.

For lipid mediator analysis, the plasma sample (10 µL) was mixed with 100 µL of methanol containing internal standard (12S‐HETE‐d8, 5 ng·mL^−1^). After centrifugation, the supernatant (50 µL) was mixed with water (200 µL) and chloroform (200 µL) and centrifuged. The organic phase was collected prior to LC‐MS injection (5 µL). A reversed‐phase column (Kinetex C8, 2.1 × 150 mm, 2.6 µm, Phenomenex, Torrance, CA) was used for chromatographic separation. For mobile phases A and B, 0.1% formic acid in water and acetonitrile were used, respectively. The flow rate was 0.4 mL·min^−1^. Column oven temperature was set at 40 °C. The gradient of mobile phase B concentration was programmed as 10% (0 min) − 25% (5 min) − 35% (10 min) − 75% (20 min) − 95% (20.1 min) − 95% (25 min) − 10% (25.1 min) − 10% (28 min). Fifteen microliters of water was co‐injected with each sample to prevent chromatographic peak leading of highly polar metabolites. Mass spectrometer parameters for positive/negative electrospray ionization mode were as follows: drying gas flow rate, 10 L·min^−1^; nebulizer gas flow rate, 2.5 L·min^−1^; desolvation line temperature, 250 °C; interface temperature, 270 °C; heat block temperature, 400 °C; collision‐induced dissociation gas, 230 kPa. The compounds were identified using the LC‐MS/MS method package for lipid mediators and LabSolutions insight software. The peak height of each compound was calculated and normalized to the peak height of the internal standard.

### Data analysis

There were some overlaps in the plasma metabolite datasets obtained using each analytical method. The overlapping metabolites include amino acids and its derivatives, nucleotides, and nucleosides. For these overlapping metabolites, the CE‐TOFMS analysis data were retained for further analysis. Then, the metabolite data from each analysis were combined. Metabolites that were detected in less than 2 animals in each group were filtered out. Data processing, normalization, and univariate and multivariate analyses were conducted using MetaboAnalyst 5.0 [18]. In the normalization process, the data were mean‐centered and divided by the standard deviation of each metabolite. Missing values were replaced by 1/5 of the minimum positive value for each metabolite. Fold change analysis, Wilcoxon rank‐sum test, and principal component analysis (PCA) were conducted with normalized metabolite data. False discovery rate (FDR) was calculated by Benjamin–Hochberg method. Differential metabolites were identified based on thresholds of fold change (< 0.77 or > 1.3), *P*‐value (< 0.05), and FDR (< 0.2).

## Results

**Fig. 1:**
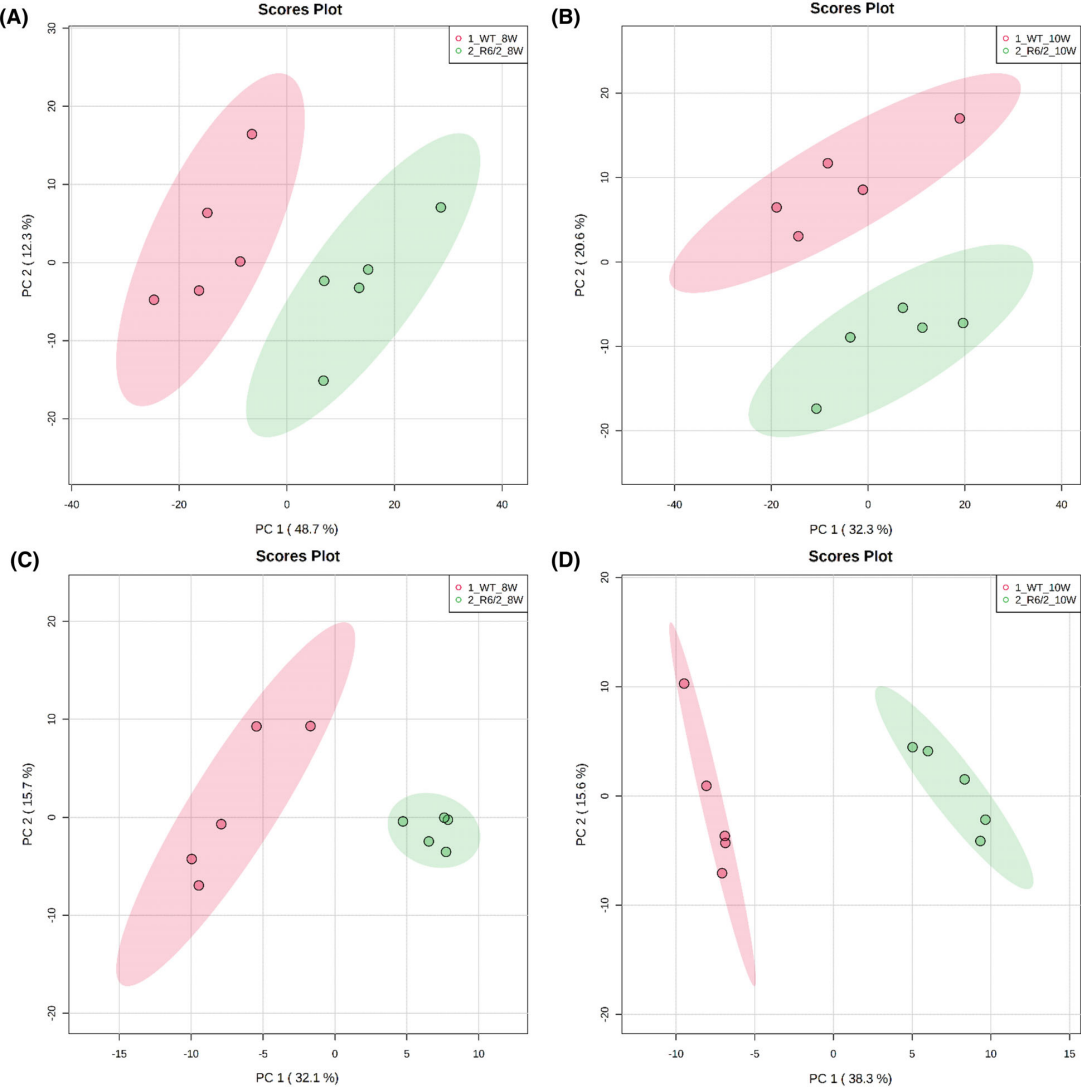
Principal component analysis scores plots of plasma and brain of R6/2 mice and WT mice: (A) plasma, 8 weeks; (B) plasma, 10 weeks; (C) brain, 8 weeks; (D) brain, 10 weeks.

In the plasma metabolomic analysis, multiple analytical techniques were applied to cover the broad range of metabolites. As a result, many metabolites were detected and identified: 202 metabolites by CE‐TOFMS analysis, 408 metabolites by DI‐LC‐MS/MS analysis using the Biocrates MxP® Quant 500 kit, and 87 metabolites by LC‐MS/MS analysis for primary metabolites and lipid mediators. The overlapped metabolites identified by different analytical methods were removed, and finally, 598 metabolites were retained for further data analysis. In the brain metabolomic analysis using CE‐TOFMS, 186 metabolites were identified and used for further data analysis. The PCA score plots (Fig. 1) show a distinct separation between the R6/2 and WT mouse groups.

**Fig. 2:**
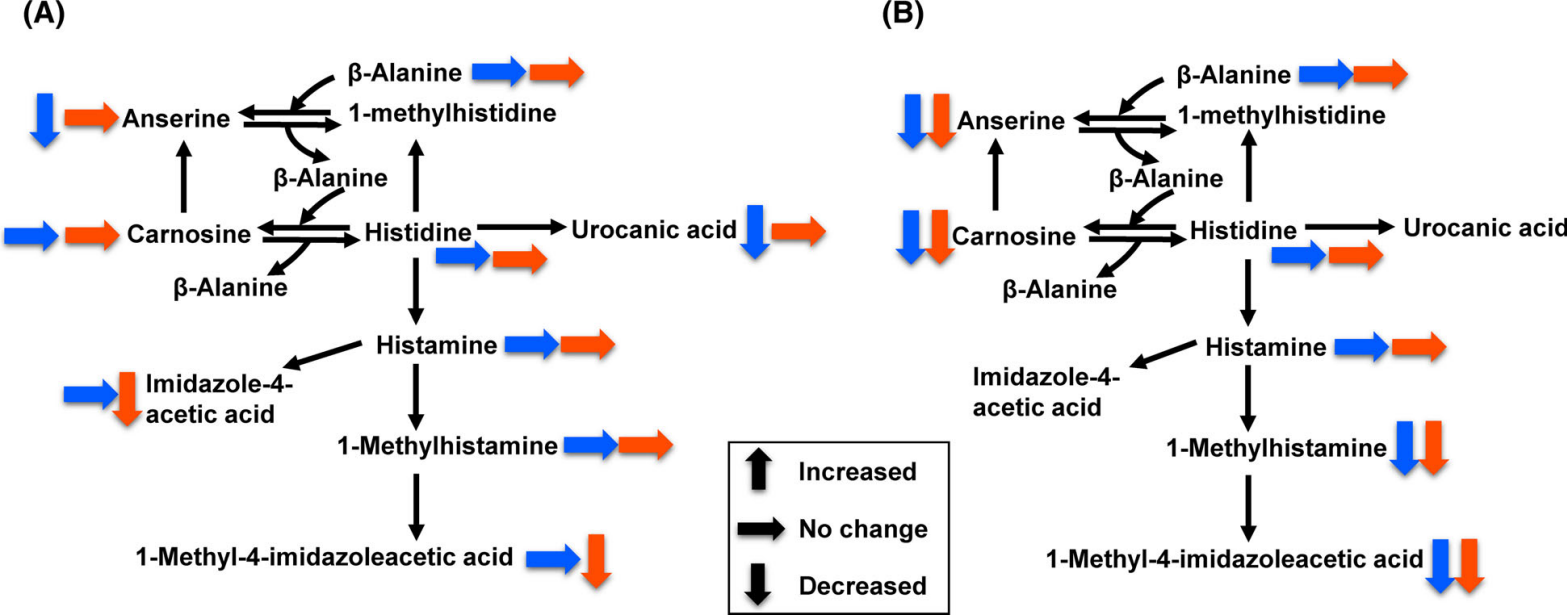
Overview of histidine metabolism in plasma (A) and brain (B) of R6/2 mice. Blue and red arrows indicate the metabolic changes of 8 weeks and 10 weeks, respectively.

**Fig. 3:**
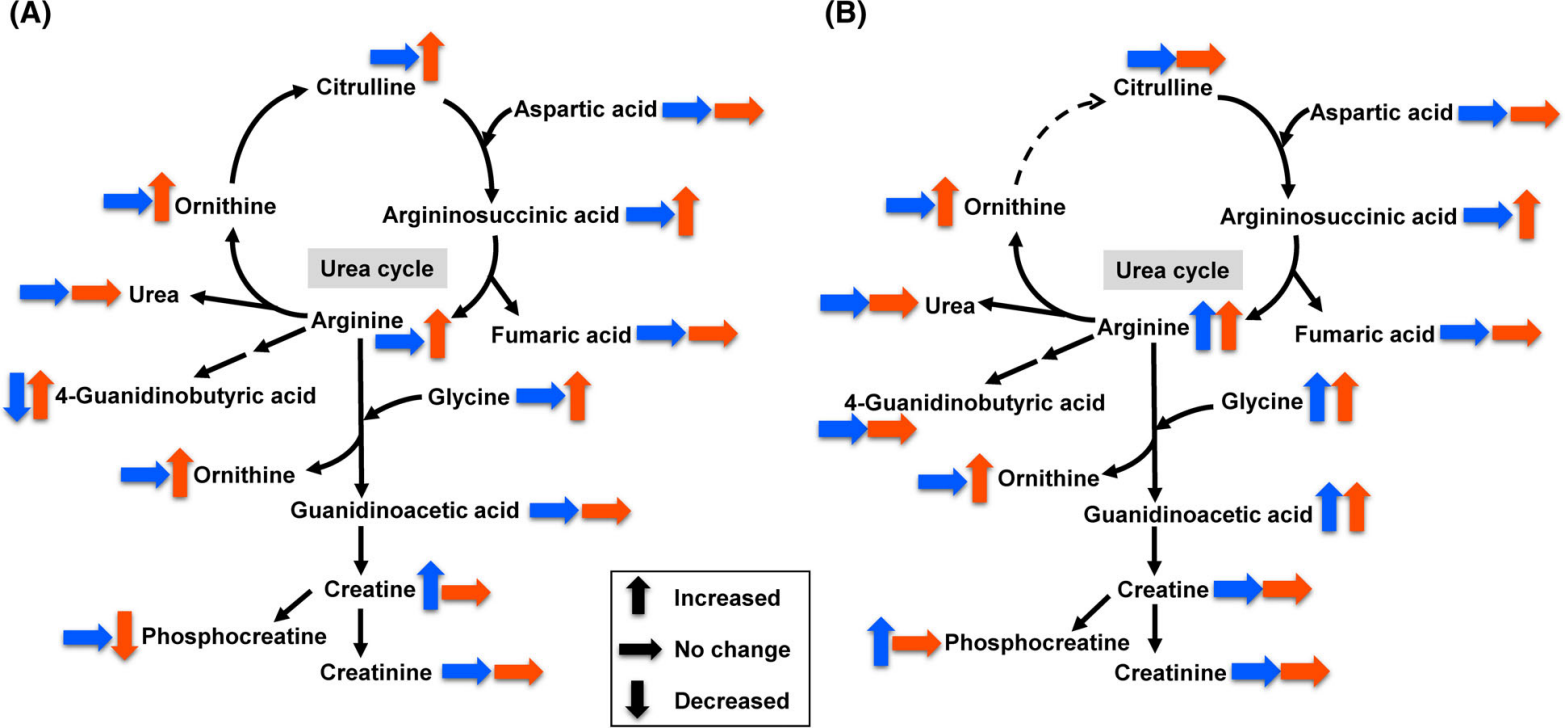
Overview of arginine metabolism in plasma (A) and brain (B) of R6/2 mice. Blue and red arrows indicate the metabolic changes of 8 weeks and 10 weeks, respectively.

**Fig. 4:**
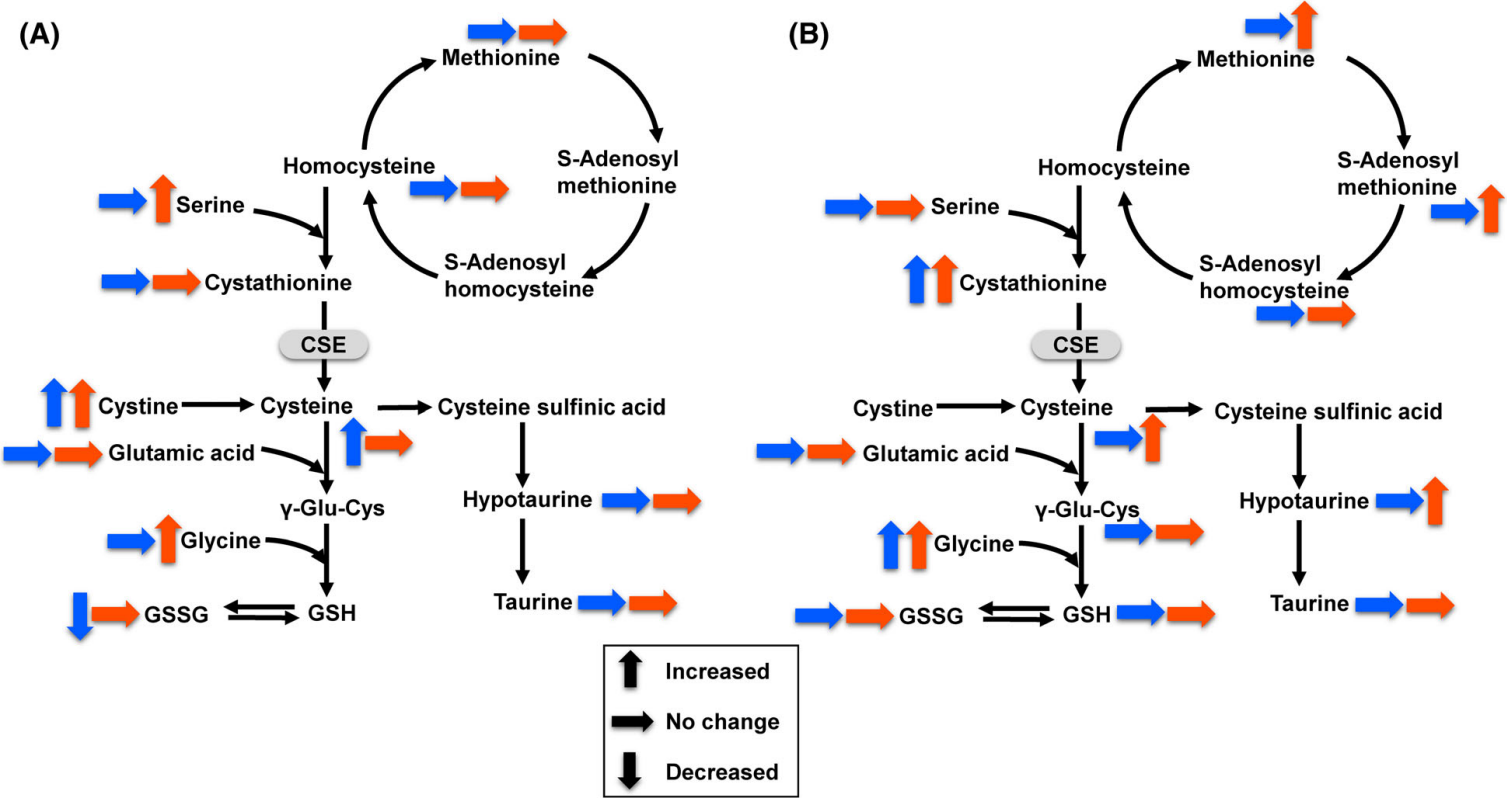
Overview of cysteine and methionine metabolism in plasma (A) and brain (B) of R6/2 mice. Blue and red arrows indicate the metabolic changes of 8 weeks and 10 weeks, respectively. CSE: cystathionine γ‐lyase.

Metabolite datasets from plasma and brain of R6/2 mice were compared with those from plasma and brain of WT mice. All the results of fold change analysis and Wilcoxon rank‐sum test are shown in Tables S1 to Tables S4. Using thresholds of fold change (< 0.77 or > 1.3), *P*‐value (< 0.05), and FDR (< 0.2), differential metabolites were identified, 260 and 77 in plasma, 31 and 45 in brain at 8 weeks and 10 weeks of age, respectively. The lists of differential metabolites were partially different between 8 weeks and 10 weeks of age, both in plasma and in brain. Many differential metabolites were related to histidine metabolism, arginine metabolism, cysteine, and methionine metabolism. The results of the fold change analysis and Wilcoxon rank‐sum test of these metabolites are summarized in Table 1. Histidine metabolism (Fig. 2) was altered in the brain of R6/2 mice at both 8 and 10 weeks of age, highlighted by the decreases in histidine‐containing dipeptides (carnosine and anserine) and histamine metabolites (1‐methylhistamine and 1‐methyl‐4‐imidazoleacetic acid). Arginine metabolism (Fig. 3) was altered in the R6/2 mice, highlighted by the increases in the urea cycle metabolites (arginine, ornithine, citrulline, and arginosuccinic acid) in plasma and brain, and guanidino compounds (guanidinoacetic acid and phosphocreatine) in brain. Cysteine and methionine metabolism (Fig. 4) was altered in the brain of R6/2 mice especially at 10 weeks of age. Alterations in specific lipid classes were also detected in the plasma of R6/2 mice (summarized in Table 2). Among the lipid species analyzed in this study, triacylglycerols, phosphatidylcholines, glycerophosphocholine, cholesterol esters, ceramides, and hexosylceramides were increased in R6/2 mice. In contrast, minor or no changes were detected in plasma acylcarnitines, lysophosphatidylcholines, sphingomyelins, hydroxysphingomyelins, and diacylglycerols.

**Table 1:** Representative metabolite profiles in R6/2 mice. HMBD, Human Metabolome Database; FC, fold change in R6/2 mice compared with WT mice; ‐, no data.

Metabolite	HMDB ID	Plasma, 8 weeks			Plasma, 10 weeks			Brain, 8 weeks			Brain, 10 weeks		
		FC	*P*	FDR	FC	*P*	FDR	FC	*P*	FDR	FC	*P*	FDR
Histidine metabolism													
Histidine	HMDB0000177	0.94	0.841	0.891	1.22	0.095	0.331	1.15	0.016	0.091	1.10	0.343	0.493
Carnosine	HMDB0000033	1.11	1.000	1.000	0.52	0.095	0.331	0.51	0.012	0.087	0.50	0.011	0.054
Anserine	HMDB0000194	0.54	0.008	0.041	1.03	0.841	0.953	0.69	0.020	0.100	0.69	0.032	0.09
beta‐Alanine	HMDB0000056	0.84	0.151	0.218	0.78	0.016	0.132	0.81	0.015	0.091	0.82	0.016	0.063
1‐Methylhistidine/3‐Methylhistidine	HMDB0000001/HMDB0000479	1.17	0.151	0.218	1.23	0.032	0.194	1.48	0.012	0.087	1.17	0.058	0.140
Urocanic acid	HMDB0000301	0.72	0.016	0.045	0.79	0.151	0.431	‐	‐	‐	‐	‐	‐
Histamine	HMDB0000870	1.36	0.690	0.775	0.86	1.000	1.000	0.88	0.674	0.869	1.03	0.841	0.908
1‐Methylhistamine	HMDB0000898	1.06	0.675	0.775	0.91	1.000	1.000	0.36	0.008	0.087	0.35	0.008	0.054
1‐Methyl‐4‐imidazoleacetic acid	HMDB0002820	1.05	1.000	1.000	0.70	0.008	0.121	0.39	0.012	0.087	0.34	0.012	0.054
Imidazole‐4‐acetic acid	HMDB0002024	0.91	0.690	0.775	0.65	0.008	0.121	‐	‐	‐	‐	‐	‐
Arginine metabolism													
Arginine	HMDB0000517/HMDB0003416	0.99	1.000	1.000	2.16	0.008	0.121	1.40	0.031	0.129	1.73	0.012	0.054
Ornithine	HMDB0000214/HMDB0003374	0.81	0.151	0.218	2.05	0.008	0.121	1.05	0.834	0.972	1.79	0.012	0.054
Citrulline	HMDB0000904	0.89	0.421	0.520	1.95	0.008	0.121	0.80	0.142	0.293	1.12	0.209	0.342
Argininosuccinic acid	HMDB0000052	1.02	0.841	0.891	1.34	0.032	0.194	1.07	0.598	0.812	1.41	0.020	0.071
Urea	HMDB0000294	0.86	0.310	0.399	0.94	0.151	0.431	0.87	0.205	0.378	0.92	0.145	0.261
Aspartic acid	HMDB0000191/HMDB0006483	1.05	0.841	0.891	1.03	0.841	0.953	0.83	0.010	0.087	0.97	0.670	0.776
Fumaric acid	HMDB0000134	0.99	1.000	1.000	0.91	0.829	0.953	0.86	0.046	0.147	0.83	0.203	0.338
Glycine	HMDB0000123	1.27	0.008	0.041	1.33	0.016	0.132	1.32	0.012	0.087	1.41	0.012	0.054
4‐Guanidinobutyric acid	HMDB0003464	0.59	0.032	0.066	3.40	0.008	0.121	0.89	0.289	0.477	1.18	0.057	0.140
Guanidoacetic acid	HMDB0000128	0.97	1.000	1.000	1.33	0.095	0.331	1.64	0.012	0.087	1.89	0.012	0.054
Creatine	HMDB0000064	1.25	0.032	0.066	1.16	0.095	0.331	1.09	0.130	0.293	1.16	0.019	0.071
Phosphocreatine	HMDB0001511	1.00	1.000	1.000	0.62	0.016	0.132	1.30	0.021	0.100	1.42	0.094	0.186
Creatinine	HMDB0000562	0.89	0.151	0.218	0.86	0.016	0.132	0.95	0.334	0.531	1.00	0.911	0.949
Cysteine and methionine metabolism													
Methionine	HMDB0000696	0.74	0.151	0.218	1.19	0.222	0.526	1.11	0.141	0.293	1.31	0.015	0.063
S‐Adenosylmethionine	HMDB0001185	‐	‐	‐	‐	‐	‐	1.14	0.036	0.135	1.30	0.012	0.054
S‐Adenosylhomocysteine	HMDB0000939	‐	‐	‐	‐	‐	‐	0.99	0.832	0.972	0.83	0.092	0.186
Homocysteine	HMDB0000742	0.95	0.548	0.653	0.90	0.421	0.715	‐	‐	‐	‐	‐	‐
Cystathionine	HMDB0000099	0.90	0.222	0.303	1.05	1.000	1.000	1.46	0.015	0.091	2.42	0.012	0.054
Cysteine	HMDB0000574	1.73	0.016	0.045	1.38	0.059	0.257	0.85	0.841	0.974	2.81	0.012	0.054
r‐Glu‐Cys	HMDB0001049	‐	‐	‐	‐	‐	‐	1.07	0.829	0.972	1.28	0.168	0.300
Glutathione (GSH)	HMDB0062697	‐	‐	‐	‐	‐	‐	1.16	0.140	0.293	2.16	0.059	0.140
Oxidized glutathione (GSSG)	HMDB0003337	0.44	0.008	0.041	0.72	0.222	0.526	0.97	0.463	0.678	0.88	0.295	0.438
Cystine	HMDB0000192	1.50	0.008	0.041	1.34	0.056	0.254	‐	‐	‐	‐	‐	‐
Hypotaurine	HMDB0000965	0.68	0.151	0.218	0.79	0.222	0.526	1.09	1.000	1.000	1.95	0.011	0.054
Taurine	HMDB0000251	1.21	0.095	0.149	1.06	0.548	0.806	1.14	0.012	0.087	1.22	0.012	0.054
Serine	HMDB0000187/HMDB0003406	1.20	0.016	0.045	1.37	0.016	0.132	0.85	0.046	0.147	0.89	0.141	0.260

**Table 2:** Overview of lipid analysis in R6/2 mice plasma.

Lipid class	Measured	Detected		Differential metabolites (increased/decreased)*	
		8 weeks	10 weeks	8 weeks	10 weeks
Triacylglycerol	242	196	194	141/0	3/0
Phosphatidylcholine	76	66	67	49/0	4/0
Glycerophosphocholine	1	1	1	1/0	1/0
Cholesterol ester	22	15	14	9/0	7/0
Ceramide	28	9	7	5/0	1/0
Hexosylceramide	34	7	6	5/0	4/0
Acylcarnitine	40	9	8	0/1	0/0
Lysophosphatidylcholine	14	9	9	0/0	2/0
Sphingomyelin	10	9	10	1/0	1/0
Hydroxysphingomyelin	5	5	5	1/0	0/0
Diacylglycerol	44	17	16	3/0	0/0

* Number of metabolite with fold change > 1.3 (increased) or < 0.77 (decreased), *P* < 0.05 and FDR < 0.2.

## Discussion

In this study, multiplatform metabolomic analysis revealed many perturbed metabolic pathways in R6/2 mouse model of HD. We found the altered histidine metabolism in the brain of R6/2 mice for the first time. In addition, changes in metabolism of arginine, cysteine, and methionine, and various lipid species were observed in R6/2 mice.

Little has been reported on the change of histidine metabolism in HD. In this study, we demonstrated the decreases in carnosine, anserine, 1‐methylhistamine, and 1‐methyl‐4‐imidazoleacetic acid in the brains of R6/2 mice (Fig. 2). Carnosine and anserine are present in mammalian skeletal muscle and brain tissues and perform various functions including pH buffering, antioxidation, metal ion chelation, and antiglycoxidation [19, 20]. In addition, there is a growing evidence indicating neuroprotective effects of these dipeptides. Recent studies have revealed the neuroprotective effects of carnosine and anserine for Alzheimer’s disease [21, 22]. Furthermore, supplementation with carnosine and anserine helps preserve verbal episodic memory in healthy elderly people and protects from cognitive decline in mild cognitive impairment [23, 24]. Various mechanisms have been proposed for the neuroprotective effects of carnosine and anserine, such as reduction of the intracellular levels of reactive species, and activation of production of brain‐derived neurotrophic factor (BDNF) and nerve growth factor (NGF) [25]. Decreased levels of carnosine and anserine observed in R6/2 mice might reflect the neurodegenerative status of HD and indicate their therapeutic potentials. Decreased levels of 1‐methylhistamine and 1‐methyl‐4‐imidazoleacetic acid were also observed in the brain of R6/2 mice. These metabolites are unique products of histamine metabolism and have been used as metabolic markers of histamine. In the brain, histamine acts as neurotransmitter and neuromodulator. The histaminergic system of the brain is involved in various physiological functions, such as modulation of sleep–wake cycle, sensory and motor functions, learning and memory [26]. Recent evidence suggests aberrant brain histamine signaling in several neurodegenerative diseases. In HD patients, postmortem study showed functional increase of histaminergic signaling in the brain [27]. Further investigations in both human and animal models are needed to clarify the relationship between brain histaminergic signaling and metabolic changes in HD brain.

Various metabolites related to arginine metabolism were altered in R6/2 mice (Fig. 3). Arginine is the intermediate in the urea cycle, which plays an important role in eliminating toxic ammonia from the body. Metabolites in the urea cycle were increased in R6/2 mouse plasma and brain, in agreement with previous reports in HD patients and animal models [28, 29, 30]. The alterations in urea cycle metabolites were more obvious at 10 weeks than 8 weeks of age, indicating that these metabolites may reflect the HD progression. Guanidino compounds derived from arginine, namely, guanidinoacetic acid and phosphocreatine, were also increased in the brains of R6/2 mice. Guanidinoacetic acid serves as a precursor of creatine, an essential metabolite involved in the energy homeostasis of nervous tissue [31]. Phosphocreatine is a reservoir of high‐energy phosphates, and both creatine and phosphocreatine play pivotal roles in maintaining the energy homeostasis in the brain [32]. Increased levels of guanidinoacetic acid and phosphocreatine in the R6/2 mouse brain may be related to the alteration of energy homeostasis in the brain, one of the key characteristics of HD [33].

The metabolism of cysteine and methionine was also altered in R6/2 mice (Fig. 4). Significant increase in brain cystathionine was observed in R6/2 mice. This result is supported by a previous report demonstrating cystathionine γ‐lyase (CSE), which converts cystathionine to cysteine, was depleted in the brains of R6/2 mice as well as HD patients [34]. Interestingly, cysteine was increased in the brain of R6/2 mice at 10 weeks of age, indicating the compensatory response to the perturbed cysteine biosynthesis. There were no significant differences in plasma cystathionine levels in R6/2 and WT mice, in agreement with a previous study demonstrating that plasma cystathionine level in HD patients was the same as those in control subjects [35]. In the R6/2 mice brain, hypotaurine was also increased at 10 weeks of age. Hypotaurine and cysteine have antioxidative and cytoprotective functions and play essential roles in responses to oxidative stress [36]. Increased oxidative stress is one of the mechanisms underlying neuronal death in HD [37]. Elevated levels of antioxidative metabolites in the R6/2 mouse brain at the later age of 10 weeks might be the response to increased oxidative stress caused by disease progression.

Alterations in lipid metabolism were also shown in the plasma of R6/2 mice. Lipids play essential roles in biological membrane formation, cell signaling pathways, and the physiological functioning of the nervous system [38, 39, 40]. Impaired lipid metabolism is one of the characteristics of neurodegenerative disorders including HD, AD, and Parkinson’s disease (PD) [41]. Previous studies have demonstrated that the increased triacylglycerols are linked to the AD pathogenesis [42]. One report also noted increased levels of plasma ceramides and hexosylceramides in PD patients [43]. In HD, mutant huntingtin protein plays a key role in dysregulation of cholesterol and fatty acid metabolism, interacting with transcription factors such as sterol regulatory element binding proteins [44, 45]. It has also been reported that mutant huntingtin protein causes direct disturbance in the stability of the phospholipid bilayer and this process is related to the mutant huntingtin aggregation [46, 47]. The results of our systematic lipid analysis demonstrated that the altered lipid profiles in R6/2 mice are specific to certain lipid classes. The increased levels of lipids such as phosphatidylcholines, glycerophosphocholine, cholesterols, ceramides, and hexosylceramides indicate the alteration of membrane biogenesis, which could affect membrane trafficking and the cell signaling cascade [48], and might be the compensatory homeostatic response for membrane disruption. The increased levels of triacylglycerols may be related to the increased levels of phosphatidylcholines, since biosynthesis of these lipids is closely related [49, 50]. The lipid classes discriminating between R6/2 and WT mice were partially different in 8 and 10 weeks of age. Increases in hexosylceramides, cholesterol esters, and glycerophosphocholine were observed both at 8 and at 10 weeks of age, indicating the chronic alteration in metabolism of these lipids. In contrast, increases in triacylglycerols, phosphatidylcholine, and ceramides in R6/2 mice were remarkable at 8 weeks rather than 10 weeks of age. Whether these changes in specific lipid classes begin at an earlier stage remains to be addressed in future research. Recent studies with HD patients proposed some potential lipid markers. Mastrokolias *et al*. reported the decreased level of serum phosphatidylcholine acyl‐alkyl C36:0 and its association with HD severity in HD patient [51]. Cheng *et al*. reported decreased levels of phosphatidylcholine acyl‐alkyl C36:0 and C34:0 and lysophosphatidylcholine acyl C20:3 in HD plasma [52]. In this study, these changes in specific phosphatidylcholines and lysophosphatidylcholine were not observed or not detected, though many phosphatidylcholines showed elevated levels in the plasma (Tables S1 and S2). Further investigations are needed to compare the lipidomic profiles in HD patients and animal models.

This study has a couple of limitations. Our findings should be interpreted with caution since this study includes only female mice with a small sample size. There are gender differences in HD both in human and in animal models, although they seem to be not so distinct [53, 54]. All biological findings in this study need to be reproduced in a larger sample set with both males and females. In addition, metabolomic investigation with broader time course as well as specific brain regions or cerebrospinal fluid can provide deeper insights into pathogenesis of HD and useful information for exploring biomarkers.

In conclusion, multiplatform metabolomic analysis revealed many perturbed metabolic pathways in R6/2 mouse model of HD. In particular, obvious perturbation of histidine metabolism in brain indicates the neurodegenerative status and altered histaminergic system. Differential metabolites related to arginine metabolism and cysteine and methionine metabolism indicate the upregulation of the urea cycle, perturbation of energy homeostasis, and increase in oxidative stress. In addition, remarkable changes in specific lipid classes indicate the dysregulation of lipid metabolism. These findings add a deeper insight into the metabolic alteration in HD and provide implications in future development of HD therapeutics.

## Conflict of interest

The authors declare no conflict of interest.

## Author contributions

MH, KW, and KM conceived and designed the experiments. MH, KW, KM, and YK performed the experiments and analyzed the data. MH, KW, KM, JT, and IM contributed to the manuscript preparation. All authors read and approved the final manuscript.

## Supporting information

**Table S1**. Plasma metabolite comparison between R6/2 mice and WT mice at 8 weeks of age.
**Table S2**. Plasma metabolite comparison between R6/2 mice and WT mice at 10 weeks of age.
**Table S3**. Brain metabolite comparison between R6/2 mice and WT mice at 8 weeks of age.
**Table S4**. Brain metabolite comparison between R6/2 mice and WT mice at 10 weeks of age.

## Data Availability

The data will be available from the corresponding author upon reasonable request.
